# Transmembrane helix prediction using amino acid property features and latent semantic analysis

**DOI:** 10.1186/1471-2105-9-S1-S4

**Published:** 2008-02-13

**Authors:** Madhavi Ganapathiraju, N Balakrishnan, Raj Reddy, Judith Klein-Seetharaman

**Affiliations:** 1 Language Technologies Institute, Carnegie Mellon University, Pittsburgh, USA; 2 Supercomputer Education and Research Centre, Indian Institute of Science, Bangalore, India; 3 Department of Structural Biology, University of Pittsburgh School of Medicine, Pittsburgh, USA

## Abstract

**Background:**

Prediction of transmembrane (TM) helices by statistical methods suffers from lack of sufficient training data. Current best methods use hundreds or even thousands of free parameters in their models which are tuned to fit the little data available for training. Further, they are often restricted to the generally accepted topology "cytoplasmic-transmembrane-extracellular" and cannot adapt to membrane proteins that do not conform to this topology. Recent crystal structures of channel proteins have revealed novel architectures showing that the above topology may not be as universal as previously believed. Thus, there is a need for methods that can better predict TM helices even in novel topologies and families.

**Results:**

Here, we describe a new method "TMpro" to predict TM helices with high accuracy. To avoid overfitting to existing topologies, we have collapsed cytoplasmic and extracellular labels to a single state, non-TM. TMpro is a binary classifier which predicts TM or non-TM using multiple amino acid properties (charge, polarity, aromaticity, size and electronic properties) as features. The features are extracted from sequence information by applying the framework used for *latent semantic analysis *of text documents and are input to neural networks that learn the distinction between TM and non-TM segments. The model uses only 25 free parameters. In benchmark analysis TMpro achieves 95% segment F-score corresponding to 50% reduction in error rate compared to the best methods not requiring an evolutionary profile of a protein to be known. Performance is also improved when applied to more recent and larger high resolution datasets PDBTM and MPtopo. TMpro predictions in membrane proteins with unusual or disputed TM structure (K^+ ^channel, aquaporin and HIV envelope glycoprotein) are discussed.

**Conclusion:**

TMpro uses very few free parameters in modeling TM segments as opposed to the very large number of free parameters used in state-of-the-art membrane prediction methods, yet achieves very high segment accuracies. This is highly advantageous considering that high resolution transmembrane information is available only for very few proteins. The greatest impact of TMpro is therefore expected in the prediction of TM segments in proteins with novel topologies. Further, the paper introduces a novel method of extracting features from protein sequence, namely that of latent semantic analysis model. The success of this approach in the current context suggests that it can find potential applications in other sequence-based analysis problems.

**Availability:**

and

## Background

Membrane proteins are a large fraction (about 30%) of all the proteins found in living organisms [1]. Knowledge of the location of transmembrane (TM) segments can be useful in narrowing down the possible tertiary structure conformations for the given protein [2-5], and in the prediction of function [6,7]. The number of TM proteins with experimentally determined structure corresponds to only about 1.5% out of about 35,000 protein structures deposited to date in the Protein Data Bank (PDB), making it desirable and necessary to predict the structure by computational sequence analysis.

All of the TM helix prediction methods make use of two fundamental characteristics: (i) the length of the TM helix being at least 19 residues so that it is long enough to cross the 30 Å thick hydrophobic core of the bilayer [8], and (ii) the TM residues being hydrophobic for reasons of thermodynamic stability in the membrane environment [9]. For transmembrane helix prediction without the use of evolutionary information, there are primarily two approaches. Simple, *explicit *methods, use a numerical scale of amino acid properties to represent primary sequence, and perform windowing and thresholding to locate long hydrophobic segments [9,10]. More advanced methods are *implicit *and treat the 20 amino acids as distinct entities, without explicit representation of their similarities, and statistically model their distribution in different topological locations of the TM proteins [11,12]. Many of the advanced methods that use statistical modeling also expect that the membrane proteins conform to the commonly observed topology of cytoplasmic-TM-extracellular. However crystal structures of a number of channel proteins have been determined recently, that do not follow the general topology [13]. For example, the KcsA potassium channel has a pore forming helix that can be confused with a TM segment [14]. In aquaporin two short TM helices have flanking loops that exit onto the same side of the membrane [15]. Owing to these deviations from the general architecture, accurate prediction of TM structure in these cases is difficult, and there is a need for prediction methods that do not restrict the "allowed" topologies of the membrane protein structure. In principle, explicit methods do not suffer from these limitations: without constraining the expected topology of the protein, they locate long hydrophobic segments and predict them to be TM helices [8,10]. However, these methods do not use additional information to overcome confusion between globular hydrophobic regions and TM segments, and suffer from low prediction accuracy [16]. One approach to reducing the errors is to use sequence profiles [17,18]. However, this results in a large number of parameters to be optimized, which is problematic given the overall little data available for training of these methods. Furthermore, evolutionary information is restricted or not available for all membrane proteins. Recently, a method was developed with a reduced number of parameters that uses evolutionary information only indirectly and incorporates structural parameters on amino acid burial derived from soluble proteins [19].

Here, we propose a different alternative approach that does not incorporate evolutionary information, only requires optimization of 25 free parameters, and is independent of topology. Based on the recent quantitative demonstration that not only hydrophobicity but also other amino acid properties, in particular aromaticity and charge, carry topological information [20,21], we developed a new method "TMpro" that derives features from several different amino acid properties to discriminate between TM and non-TM segments. TMpro uses a classification algorithm (an artificial neural network, a hidden Markov model or a linear classifier) to learn these features for TM prediction independent of the membrane topology. The framework for sequence representation, feature extraction and data processing for prediction used in TMpro is analogous to the framework developed previously for classification of secondary structure elements based on *latent semantic analysis *used for information retrieval and summarization in natural language processing (described in detail in ref. [22]). In this approach, the secondary structure or here TM segments are treated as equivalent to text-documents, and are represented as *bag-of-words *in terms of the underlying vocabulary. For TMpro, the vocabulary consists of {positive charge, negative charge, neutral charge, aromatic, aliphatic, ...}. Segment/document similarity is computed based on the frequencies of occurrence of the "words" in the segment/document. The method is tested on established benchmark as well as more recent data sets, and is found to perform significantly better than other methods that also do not use evolutionary information in segment accuracies and similar to those that do use evolutionary information.

Previous benchmark analysis on a dataset of proteins for which high-resolution crystallographic information was available at the time [16] and other similar comparative evaluations [23-26] have shown that TMHMM [27] is one of the best methods for TM helix prediction from sequence alone. TMHMM is thus a widely accepted method to analyze large datasets and also to study specific proteins [1,28]. Even though the benchmark server [29] uses a somewhat outdated dataset for testing, it is an excellent resource to quantitatively compare TM helix prediction methods. Using the benchmark server, TMpro achieves 30–50% reduction in segment error rate in comparison to the top-performing single sequence methods TMHMM, SOSUI [30], DAS-TMFILTER [31,32] and ranks second best in segment accuracy, closely following PHDpsihtm08, a method that uses evolutionary information [18]. Although accuracy, rather than error rate, is a more common measure of prediction performance it must be noted that the latter provides an absolute quantification of improvement. While the significance of a 5% increase in accuracy varies relative to the initial accuracy level, a 5% decrease in the error rate indicates the same amount of significance irrespective of the initial error rate – namely, a 50% error reduction resulting from accuracy increase from 80% to 90% is as significant as that resulting from only a 5% increase in accuracy from 90% to 95% which yields the same reduction in error rate of 50%. We also evaluated TMHMM on most recent available data sets, MPtopo [33] and PDBTM [34].

## Results and discussion

### Amino acid properties other than hydrophobicity are also predictive of TM structure

To estimate the predictive capacity of different properties of amino acids, we used our protein sequence representations according to the groupings of amino acids by different properties described in Methods, and applied the TMHMM architecture to these reduced representations. We used the publicly available model parameters of TMHMM (version 1) as-is [35], and tested the prediction accuracy on the set of 36 high resolution proteins (Methods). First, we obtained the accuracies with the original representation of 20 amino acids as a control. Next, the possible observations in each state are collapsed from 20 to 2 possibilities for polarity (polar or nonpolar), 3 for charge, 3 for aromaticity, 3 for size and 5 for electronic properties (Methods). To illustrate the procedure using polarity as an example, the probability mass of all polar amino acids is summed to yield the probability of observation of a polar residue in that state, as given in Equation 1.

$$\begin{array}{ll} P(0|q)=\sum_{o}P(o|q) & o\in \{A,D,E,F,G,I,L,M,P,V,W\}\\ P(1|q)=\sum_{o}P(o|q) & o\in \{C,H,K,N,Q,R,S,T,Y\} \end{array}$$

where, *P*(*x *| *q*) refers to the probability of observing *x *while in state *q*; observation *0 *refers nonpolar residue, and observation *1 *refers to a polar residue. The primary sequence of the test set proteins is similarly mapped to a sequence of 0's and 1's depending on whether the residues are nonpolar or polar respectively. TM helices are then predicted for the mapped sequences with the TMHMM models modified by Equation 1.

The results are shown in Table 1. When the 20 possible amino acids are collapsed drastically to only the 2 possible property values, polar or nonpolar, the prediction accuracy of TM segments (segment F-score) is still at ~59%. In other words, the 2-valued polarity (or hydrophobicity) of the residues contains ~64% of the information compared to that given by 20-amino acid representation. Surprisingly, even more remarkable results were obtained with the other representations: the segment F-score of TM prediction with aromaticity property (3-valued scale) and electronic property (5-valued scale). Table 1 shows that for both of these properties, the amount of predictive information contained by the collapsed representation of primary sequence is close to 92% of the information encoded in the full spectrum of the 20 amino acids. The residue accuracy Q_2 _is in a similar range – the single property representations contain 80–94% of the information encoded by the full list of 20 amino acids.

**Table 1:** Accuracy of TM prediction with TMHMM architecture but using property representations of residues in comparison to full 20 letter amino acid representation.

1	2	3	4	5	6
Sequence representation	Number of Symbols	Segment F-score	Segment F-score as % of that with amino acid representation	Q_2_	Q_2 _score as % of that with amino acid representation

Amino acids	20	92	-	81	-
Polarity	2	59	64%	65	80%
Aromaticity	3	84	91%	74	91%
Electronic property	5	85	92%	76	94%

The TMHMM architecture, with model parameters corresponding to version 1  was used for TM helix prediction. Note that all other comparisons used TMHMM 2.0. The first row of results labeled Amino acids corresponds to this "rewired" TMHMM. Accuracies are computed locally, with metrics corresponding to those defined in [2]. F-score is the geometric mean of the segment recall (Qobs,htm) and segment precision (Qprec,htm). Next row, marked 'polarity' corresponds to the TMHMM when the observation probabilities of 20 amino acids are grouped to form 2 observations, namely polar and nonpolar. Columns 4 and 6 give what percentage contribution is made by polarity representation in comparison to that of 20 amino acid representation. For example, polarity representation achieves 59% F-score, which is 64% of the 92% segment F-score achieved by amino acid representation. Results for aromaticity representation and electronic property representation are given similarly in the next two rows.

These results demonstrate that even with a rudimentary representation of the amino acid sequence as polar/nonpolar, aromatic/aliphatic/neutral, electron donor/acceptor/neutral, significant prediction accuracy can be achieved. This observation strongly validates the hypothesis that amino acid properties other than hydrophobicity/polarity alone have predictive capacity. This prompted us to test if we can exploit this fact to develop a new TM prediction algorithm that makes use of this additional information.

### Analogy to latent semantic analysis

A major challenge in TM helix prediction is the danger of overfitting because of the small amount of available training data, even if only few features are used in model development. Text document classification suffers from the same difficulty, although in the human language technologies domain largely because of the hundreds of thousands of different words in the vocabulary. Latent semantic analysis is a method successfully used for text document summarization to address this problem. In latent semantic analysis, similarities between documents or sentences are captured by studying the underlying word distributions – the order of appearance of the words is not preserved but the overall frequencies are accounted for [36]. Singular value decomposition (SVD) and selection of the "top energy" (or "high variance") words is used to reduce the noise in the data. Here, we propose to use latent semantic analysis in direct analogy to its application in language by capturing amino acid property distributions in TM segments analogous to word distributions in text documents. To this end, the windowed segments of protein sequences are represented as *bag-of-words*, where words here are the different amino acid properties – namely, polar, non-polar, charged positive, charged negative, and so on. Although the number of words in the vocabulary of TM protein structure is very small (only 10 are considered in this work), singular value decomposition and discarding of low energy dimensions is still necessary to overcome overfitting of the statistical models to the very small training data. Discarding of highly specific feature dimensions ensures over fitting of statistical models does not happen. We verified the benefit of using latent semantic analysis as a feature reduction method by comparing classification using the full set of features and the latent semantic analysis derived ones (see section "Need for Latent Semantic Analysis", below).

### Separability of the feature vectors

**Figure 1:**
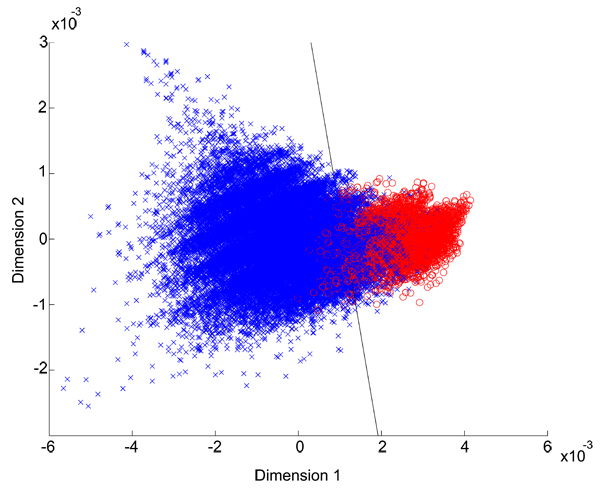
**Classification of protein feature vectors of the completely-membrane or completely-nonmembrane type**: Figure shows the data points of the training set, and linear classifier learnt from this data. The first two dimensions of the features after principal component analysis are shown in the scattergram. It may be seen that even a simple linear classifier can separate out a large fraction of the data points into the correct class.

First, we estimated the separability of the feature vectors derived from latent semantic reduction of amino acid property features. Figure 1 shows a scattergram of the first two dimensions against each other of features derived with window size 16. Data corresponding to completely non-TM type are shown with a blue '+' marker and those corresponding to completely TM type are shown with a red 'o' marker. A linear classifier learnt using Fischer's discriminant over these data points is also shown (black line). It can be seen qualitatively that although there is a region of confusability, a large number of data of either class fall in the non-confused region. We can use the linear classifier to estimate the separability of the feature sets. Of the feature vectors originating from completely-TM or completely non-TM windows of the training data, only 7% are misclassified. When all the feature vectors of the training set including those with *mixed *label are classified, only 15% of the features are misclassified, indicating that there is a good separability of the TM features from non-TM features. In TMpro, we used a neural network to learn the boundary between these feature vectors. When a smaller window size of only 6 residues is used, features corresponding to TM and non-TM are not separable with a boundary. We therefore used a hidden Markov model that can capture gradual variation in the features along the sequence. The TMpro feature vectors combined with the linear classifier, the HMM and the NN classifier, will be referred to in the following as TMpro LC, TMpro HMM and TMpro NN, respectively.

### Benchmark analysis of transmembrane segment prediction in membrane proteins

In order to compare the performance of the three different implementations of TMpro to previous work we used the TMH benchmark web server for evaluations [16]. We trained our models with the same data set as had been used for training TMHMM, namely the set of 160 proteins. The data set used for evaluation is the set of 36 proteins (called high-resolution data set) from the benchmark analysis paper [16], referred to as dataset 1, below.

We performed the evaluations by submitting the predictions on the benchmark evaluation server [29]. The predictions on alpha helical membrane proteins are evaluated by the following set of metrics [16]: Q_ok _is the percentage of proteins whose membrane segments are all predicted correctly. Segment recall (Q^obs,htm ^on benchmark server) is the percentage of experimentally determined (or 'observed') segments that are predicted correctly. Segment precision (Q^pred,htm ^on benchmark server) is the percentage of predicted segments that are correct. The residue accuracy Q_2 _refers to the percentage of residues that are predicted correctly. We also computed the F-score, which is the geometric mean of segment level recall and precision (Q^obs,htm ^and Q^pred,htm^). Since recall and precision can each be increased arbitrarily at the expense of the other value, the two metrics when seen independently do not reflect the strength of the algorithm. Hence, the geometric mean of the two, (effectively the point where the two measures are expected to be equal) is used as the metric.

The evaluation of TMpro (LC), TMpro (HMM) and TMpro (NN) by the benchmark server (on dataset 1) is shown in Table 2, in comparison to that of TMHMM [16]. All three implementations of TMpro show improvements over TMHMM results. Even the simple linear classifier yields a 4% increase in the F-score, with an "*even increase*" in both the segment recall and precision. The HMM model improves the Q_ok _compared to the linear classifier. While the F-score remains the same, there is an imbalance between recall and precision. Although Q_ok _in both TMpro (LC) and TMpro (HMM) is lower than in TMHMM, the segment level accuracies are improved in both these methods. TMpro (NN) shows the highest improvement in Q_ok_. The results obtained with the NN method yield a Q_ok _of 83% (a 12% increase over TMHMM). A high value of Q_ok_, which is the most stringent metric at the segment level, indicates that the TMpro NN achieves very good prediction of TM helices. This value of Q_ok _is higher than those achieved by any of the methods that have been evaluated by [16] excepting HMMTOP (which uses the entire test set of proteins in training, as opposed to only 7 proteins of the testing set are used in training TMpro and TMHMM), and PHDpsihtm08 [37] (which uses evolutionary information and a complex model with hundreds of model parameters). The segment F-score reaches 95% with an even balance between segment recall and precision. This segment accuracy represents a 50% reduction in error rate as compared to TMHMM, which is the best method not using evolutionary information evaluated in the benchmark analysis [16]. In other words, 10% of errors in the segments missed or over-predicted by TMHMM, half of those difficult segments are correctly predicted by TMpro. TMHMM misclassifies 3 proteins as soluble proteins, in contrast to TMpro which does not misclassify any. The results of all the methods evaluated in benchmark are shown in Table 3.

**Table 2:** Comparison of TMpro methods with that of TMHMM on high resolution data set of 36 proteins from benchmark analysis.

	Method ↓	Q_ok_	Segment F Score	Segment Recall	Segment Precision	Q_2_	# of TM proteins misclassified as soluble proteins
**High resolution proteins (36 TM proteins)**							

1	TMHMM	71	90	90	90	80	3
2	TMpro LC	61	94	94	94	76	0
3	TMpro HMM	66	95	97	92	77	0
4	TMpro NN	83	96	95	96	75	0

**Without SVD**							

5	TMpro NN without SVD	69	94	95	93	73	0

It can be seen that TMpro achieves high segment accuracy (F-score) even with a simple linear classifier. For a description of evaluation metrics see caption of Table 3. To demonstrate the requirement of singular value decomposition of features, the results obtained by directly using property count features without employing SVD are shown in the last row. It may be seen that the results are slightly poor compared to standard TMpro.

**Table 3:** Evaluation results on the benchmark data set.

	Protein-level accuracy			Per-segment accuracy			Per-residue accuracy
**Method**	**Q_ok_**	**False Positives**	**False negatives**	**Qhtm Fscore**	**Qhtm %obs**	**Qhtm %prd**	**Q2**

PHDpsihtm08	84	2	3	98	99	98	80
**TMpro**	**83**	**14**	**0**	**95**	**95**	**96**	**73**
HMMTOP2	83	6	0	99	99	99	80
DAS	79	16	0	97	99	96	72
TopPred2	75	10	8	90	90	90	77
TMHMM1	71	1	8	90	90	90	80
SOSUI	71	1	8	87	88	86	75
PHDhtm07	69	3	14	82	83	81	78
KD	65	81	0	91	94	89	67
PHDhtm08	64	2	19	76	77	76	78
GES	64	53	0	93	97	90	71
PRED-TMR	61	4	8	87	84	90	76
Ben-Tal	60	3	11	84	79	89	72
Eisenberg	58	66	0	92	95	89	69
Hopp-Woods	56	89	0	89	93	86	62
WW	54	32	0	93	95	91	71
Roseman	52	95	0	88	94	83	58
Av-Cid	52	95	0	88	93	83	60
Levitt	48	93	0	87	91	84	59
A-Cid	47	95	0	89	95	83	58
Heijne	45	92	0	87	93	82	61
Bull-Breese	45	100	0	87	92	82	55
Sweet	43	84	0	86	90	83	63
Radzicka	40	100	0	86	93	79	56
Nakashima	39	90	0	85	88	83	60
Fauchere	36	99	0	86	92	80	56
Lawson	33	98	0	82	86	79	55
EM	31	99	0	84	92	77	57
Wolfenden	28	2	39	52	43	62	62

Performance of methods other than TMpro were originally reported in Protein Science [16] and are reproduced here with permission from Cold Spring Harbor Laboratory Press, Copyright 2002. TMpro values in comparison to these published values are returned by the benchmark web server [29] when TMpro predictions are uploaded. The columns from left to right show: method being evaluated; Protein level accuracies: Q_ok_, which is the percentage of proteins in which all experimentally determined segments are predicted correctly, and no extra segments are predicted; that is, there is a one to one match between predicted and experimentally determined segments. False positives, which is the percentage of globular proteins that are misrecognized as membrane proteins. False negatives, which is the number of membrane proteins that are misclassified as soluble proteins because no TM segment is predicted in the protein. In segment level metrics are shown segment F-score which is the geometric mean of Recall and Precision, Recall (Q^htm,%obs^, percentage of experimentally determined segments that are predicted correctly), Precision (Q^htm,%pred ^percentage of predicted segments that are correct). Q_2 _is the residue level accuracy when all residues in a protein are considered together, and the Q_2 _value for the entire set of proteins is the average of that of individual proteins. See [16] for further details on these metrics.

### Performance on recent MPtopo and PDBTM data sets

The benchmark analysis described in the previous section is useful in comparing the TMpro method with other well accepted methods, but the evaluation data set does not include recently determined membrane protein structures. We therefore computed the accuracies achieved by the TMpro on two recent data sets, MPtopo and PDBTM. In order to allow for a fair comparison with TMHMM, the training set was kept the same as that used for TMHMM 2.0, namely the set of 160 proteins. 12 out of 191 proteins of PDBTM and 16 out of 101 proteins of MPTOPO are contained in the training set. Since TMpro (NN) gave the best prediction results in the benchmark analysis, we only studied TMpro (NN) further. **In this and the subsequent sections, we henceforth refer to TMpro (NN) as TMpro**. In the evaluation of TMpro performance on more recent data, we also compared our predictions with two other algorithms that do not use evolutionary profile: SOSUI [30]and DAS-TMfilter [31,32].

The results of the comparison between TMpro, TMHMM, SOSUI, DAS-TMfilter are shown in Table 4. As can be seen, TMpro achieves a 2–3% increase in segment F-score in comparison to TMHMM, 4–6% in comparison to SOSUI and 3–5% in comparison to DAS-TMfilter. Thus, we conclude that amino acid properties used in conjunction with latent semantic analysis and neural network classifier are highly predictive of TM segments on the two recent data sets.

**Table 4:** Evaluation of TMHMM, SOSUI, DAS TMfilter and TMpro NN prediction performance on PDBTM non-redundant set and MPtopo high resolution set.

			**F**	**Qhtm**	**Qhtm**		
					
	**Method**	**Q_ok_**	**Score**	**%obs**	**%prd**	**Q2**	**Confusion with soluble**
**PDBTM (191 proteins, 789 TM segments)**							

1	TMHMM	68	90	89	90	84	13
2	SOSUI	60	87	86	87		17
3	DAS TMfilter	62	90	90	91	85	10
4	**TMpro NN**	57	93	93	93	81	2

Without Singular Value Decomposition (SVD)							

5	TMpro no SVD	57	91	93	90	81	

**MPtopo (101 proteins, 443 TM segments)**							

6	TMHMM	66	91	89	94	84	5
7	SOSUI	68	89	91	87	82	7
8	DAS TMfilter	66	88	87	90	78	5
9	**TMpro NN**	60	93	92	95	79	1

For description of columns, see caption of Table 3. Q^htm,%obs ^and Q^htm,%pred ^have been computed per-protein and averaged over all the proteins. Last column shows the number of membrane proteins that have been mistaken as soluble proteins.

### Need for latent semantic analysis

We addressed the question whether or not the latent semantic analysis is really needed, because the TM structure vocabulary is much smaller than the word vocabulary in language. To this end, a different neural network was trained with 10 dimensional input vectors wherein the SVD step was removed, and the 10-word input dimensions were connected directly to the neural network. Segment accuracies were found to be about 2% lower for the F-score segment accuracy for the benchmark and PDBTM data sets (see Tables 2 and 4). We conclude that although the TM structure input vocabulary is small, the SVD step is useful for high accuracy TM segment prediction. We attribute this advantageous effect to the small available training data in this field.

### Confusion with globular proteins

The benchmark server provides a set of 616 globular proteins also for evaluation. Although classification of proteins into globular and TM types is a problem fundamentally different from predicting the sequential positions of TM helices in TM proteins and the use of TM helix prediction methods to differentiating between TM and non-TM proteins is a misuse of these methods, it is still a useful exercise in estimating to what degree hydrophobic helices in soluble proteins are confused to be TM helices. We found that 14% of the globular proteins in the dataset provided on the benchmark server are confused to be that of membrane type by TMpro. However, it is to be noted that all the methods that have lower confusion with globular proteins also miss many membrane proteins and wrongly classify them to be of globular type (see Table 3). TMpro misclassifies only 1 of the MPTopo proteins as soluble protein, whereas TMHMM and DAS-TMfilter misclassified 5 TM proteins and SOSUI misclassified 7 TM proteins as soluble proteins. In the PDBTM set, TMpro misclassifies only 2 proteins as soluble proteins as compared to 13 proteins by TMHMM and 17 proteins by SOSUI and 10 proteins by DAS-TMfilter that were misclassified (Table 4).

### Application to specific proteins

**Figure 2:**
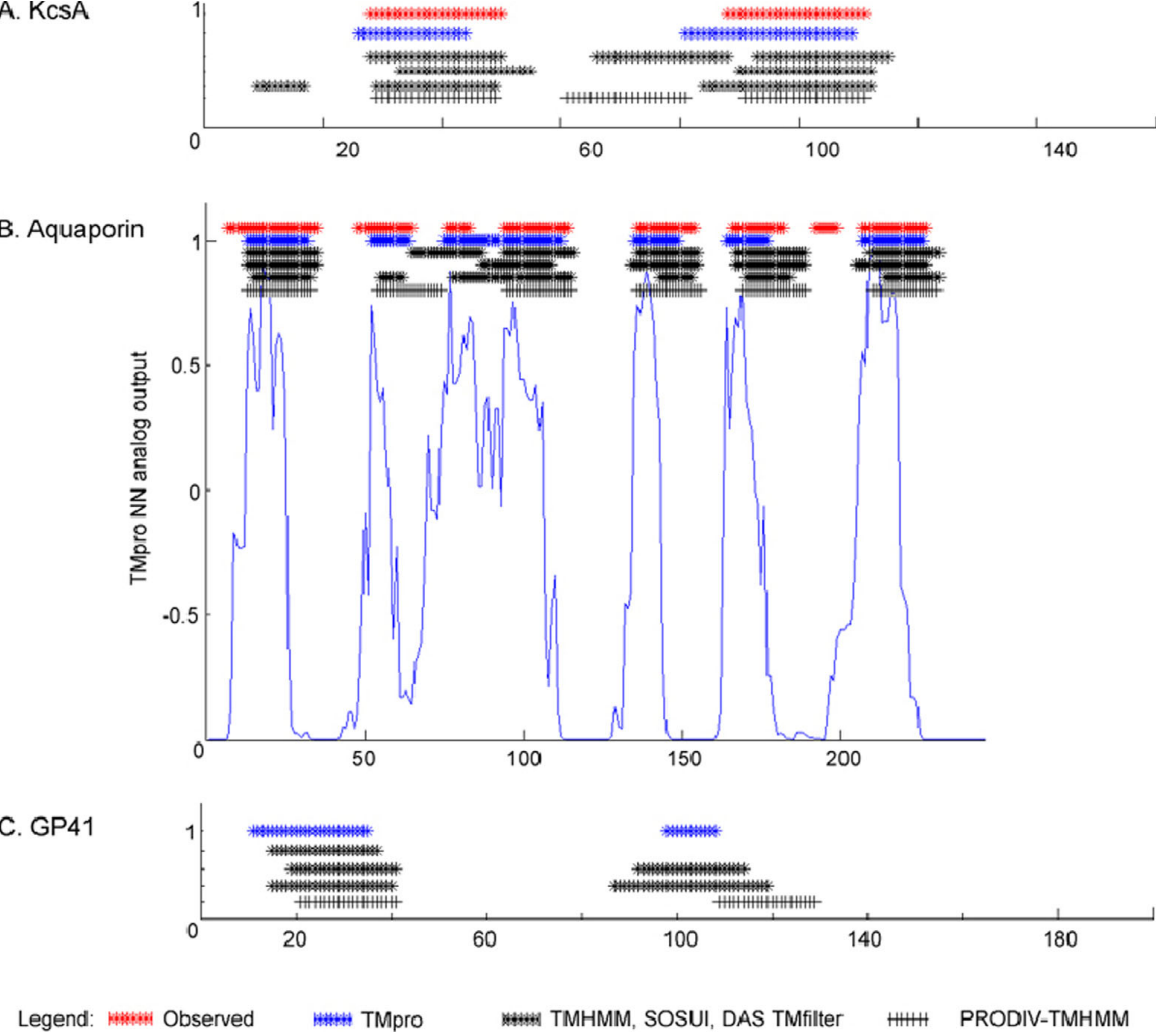
**Predictions of membrane segments in specific proteins**: (A) KcsA potassium channel, (B) aquaporin and (C) human immunodeficiency virus glycoprotein GP41.  Observed segments (red) and predicted segments by TMpro, TMHMM version 2.0, SOSUI, DAS-TMfilter and PRODIV-TMHMM are shown in this order. Experimentally observed segments are not known for GP41.  For aquaporin, the analog output of TMpro neural network is also shown. This representation reveals that the 3rd predicted helix, which is unusually long, shows a relative minimum in its center. Although it is not sufficient for automatic prediction, it does indicate the possibility of a separating loop, as is observed experimentally.

In the above sections, we have demonstrated that without using evolutionary information, without restricting the membrane topology and with only using 25 free parameters, the TMpro approach results in very high accuracies in TM structure prediction of TM proteins with known topology. We believe that these features will make TMpro particularly useful in future predictions of TM helices in proteins from novel families and with novel topologies. Although substantiating this claim quantitatively will require solving new membrane protein structures, we would like to present three specific examples to qualitatively illustrate the potential strengths and weaknesses of this method. Figure 2 shows the predicted TM segments of the KcsA potassium channel (PDB ID 1BL8, [14]), the aquaporins (represented by PDB ID 1FQY [15]) and the human immunodeficiency virus (HIV) envelope glycoprotein gp41 (structure unknown). TMpro results are compared to those from TMHMM, DAS-TMfilter, SOSUI as representatives of single-sequence methods, and PRODIV-TMHMM as a representative of a multiple-sequence alignment-based method.

(i) KcsA potassium channel: In contrast to the general topology of membrane proteins which have a membrane segment completely traversing from the cytoplasmic (cp) to extracellular (ec) side or vice versa, resulting in a ...cp-TM-ec-TM-cp... topology, the KscA potassium channel has a short 11-residue pore-forming helix (ph) and an 8-residue pore-forming loop (pl) that are surrounded by TM helices of a tetrameric arrangement of 4 chains. The loops on either side of this short helix exit onto the extracellular side of the membrane, giving the protein the topology of "cp-TM-ec-ph-pl-ec-TM-cp". The predictions of the TM segments in the KcsA potassium channel are shown in Figure 2A. TMHMM incorrectly predicts part of the pore-forming helix and a part of the extracellular loop together as a TM segment while TMpro correctly predicts 2 TM segments, matching with the observed segments. SOSUI also correctly predicts only 2 TM segments while DAS-TMfilter predicts 3 segments. The evolutionary method PRODIV-TMHMM predicts 3 segments incorrectly.

(ii) Aquaporins. Aquaporins also deviate from the ...cp-TM-ec-TM-cp... topology in that they have two short TM helices (about half the length of a normal TM helix) which are very distant in primary sequence but are close in the 3D structure to form what looks like a single TM helix in a back to back orientation of the two short helices. In this highly unusual topology, the two short helices are of the type cp-TM-cp and ec-TM-ec. The TM helix predictions are shown in Figure 2B. None of the methods compared can correctly predict both short TM helices, including TMpro. Of the observed eight TM helices, TMpro, TMHMM and DAS-TMfilter predict 6 while SOSUI predicts 5. TMpro and DAS-TMfilter both predict an unusually long helix that connects TM segments 3 and 4. Although this prediction is wrong, both the methods provide some evidence for the separation of the two TM helices: DAS-TMfilter gives a text output that there is a possibility of two helices; in the analog output of TMpro NN there is a minimum at the position of the loop. In contrast, PRODIV-TMHMM is not able to infer the two short helices. However, it does show a better alignment of the other predicted helices with the observed locations.

(iii) HIV glycoprotein gp41. The topology of gp41 is not known, and there is significant debate over the nature of the putative TM segments [38]. The TM helix predictions are shown in Figure 2C. TMpro predicts two TM segments with high confidence; one of them is the known fusion peptide, which constitutes a TM helix during HIV fusion with the host cell. Of the other methods compared (TMHMM, DAS-TMfilter, SOSUI, PRED-TMR, HMMTOP), only DAS-TMfilter and SOSUI predict two TM segments – the other methods do not predict the TM helix at all and predict the fusion peptide as the only TM segment. Such a prediction is incompatible with the experimental evidence that gp41 is a TM protein, while the fusion peptide is buried in a hydrophobic, but soluble non-TM core.

## Conclusion

All TM helix prediction methods make use of the fact that the propensities of amino acids are characteristically different in TM helices as compared to soluble portions. The most successful methods incorporate very restrictive topologies into complex statistical models of amino acid propensities. These models use hundreds to thousands of free parameters but are trained with the limited data set available today. In this paper, we describe a method TMpro, which uses only 25 free parameters, does not use topology restrictions and does not require evolution information for success. The method is based on using novel amino acid property reduced vocabulary features beyond traditional use of hydrophobicity in conjunction with application of methods borrowed from text document classification. TMpro diplays high (>90%) segment accuracy consistently across benchmark and more recent TM protein datasets and is therefore well suited for segment level prediction. TMpro also shows promise in predicting TM segments in membrane proteins with unusual topology which are difficult to recover by other methods. The current Q_ok _values indicate that the method has still room for improvement, which could be obtained by reducing the false positive error rate, work that is currently underway.

Further, the paper introduces a novel method of extracting features from protein sequence, namely that of latent semantic analysis model. Most methods for transmembrane helix prediction capture amino acid propensities through single numbers (as in hydrophobicity scales) or probability distributions (as in hidden Markov models). Here, we transform the training data into a new feature space, and learn a boundary that separates the transmembrane features from non-transmembrane features using a neural network model. The success of this approach suggests that it can find potential applications in other sequence-based analysis problems.

## Methods

### Data sets

The training set is the set of 160 proteins [11] used to train TMpro and for comparison TMHMM version 2. Three different data sets of alpha helical TM protein sequences with high resolution information are used for evaluation: (1) the set of high resolution proteins from the benchmark evaluations by Chen et. al [16]. (2) TM proteins with high resolution information from the MPtopo data set consisting of 443 TM segments in 101 proteins [33]. (3) A PDBTM dataset downloaded in April 2006 which contains all transmembrane proteins with 3D structures from the PDB determined to that date [34]. PDBTM provides a list of non-redundant subset of the set of alpha-helical TM proteins. Non-redundant is defined as having sequence identity less than 40% [34]. Chains corresponding to this non-redundant list were extracted from the complete set, resulting in 191 proteins consisting of 789 TM segments.

Instead of formulating a 3-class labeling scheme referring to "inside", "outside" and "membrane", we used a 2-class labeling scheme "TM" and "non-TM", where the data labels corresponding to inside and outside are mapped to a single state 'non-TM'.

### Approach

**Figure 3:**
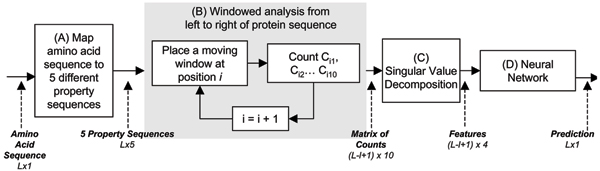
**Block diagram of TMpro prediction. **Primary sequence of protein (amino acid sequence) is input to the system. (A) maps it to 5 amino acid property se-quences. The output has size 5 x L (rows x columns). These 5 sequences form input to (B) which performs windowed analysis, and outputs a matrix of counts of 10 properties (C1 to C10) for each window position. This output has the size 10xL-l+1. The outputs from (B) for all proteins are collected together and singular value decomposition is performed by C. During testing phase, an SVD approximation is performed for the matrix of a single test protein. The output of this block (C) forms the final features used by neural network. (D) Features are evaluated by the NN model and a 2-state prediction is output for each residue (TM, non-TM). An analog value also is output ranging from -1 to 1 that indicates the closeness of the feature to non-TM or to TM correspondingly.

The proposed approach is to find TM segment features derived from amino acid property representations and then use a suitable classification or statistical modeling method to achieve better accuracy in TM prediction (Figure 3).

(1) To begin with, the primary sequence which is originally in terms of the 20 amino acids, is decomposed into five different primary sequences, each one representing one property of the amino acids, namely polarity, charge, aromaticity, size and electronic property.

(2) These property label sequences are then studied in a moving window.

(3) The feature space is reduced by singular value decomposition.

(4) As opposed to a simple threshold yielding a linear boundary between TM and non-TM features, an advanced statistical model is used to separate the features in the two classes by a nonlinear boundary. A neural network (NN) is used to classify the reduced dimension features as TM and non-TM, while a hidden Markov model (HMM) is built independently to capture the sequential nature of TM and non-TM features. The HMM architecture used here is a simpler one and therefore less restrictive compared to the models of TMHMM or HMMTOP [12,27].

(5) The prediction labels output by NN and HMM are arrived at independently.

### Data preprocessing

#### Step 1: Protein sequence representation

**Figure 4:**
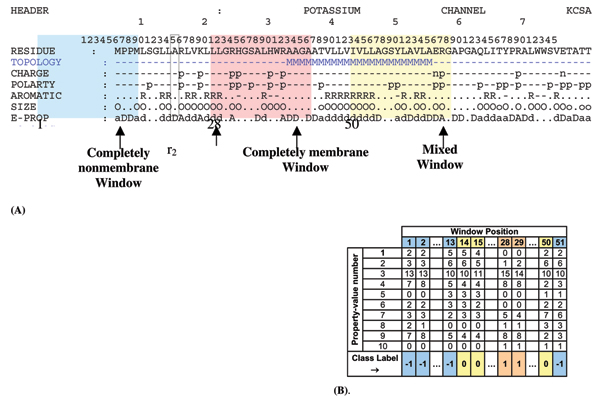
**(A) Data preprocessing and feature extraction**: A sample sequence is shown with its property annotations. Line (1) header of the protein (potassium channel Kcsa). Line (2) primary amino acid sequence. Line (3) Topology: nonmembrane ‘-’ and membrane ‘M’. Line (4) Charge: posi-tive ‘p’, negative ‘n’ and neutral ‘-’. Line (5) Polarity: polar ‘p’ and nonpolar ‘-’. Line (6) Aro-maticity: aromatic ‘R’, aliphatic ‘-’ and neutral ‘,’. Line (7) Size: small ‘.’, medium ‘o’ and large ‘O’. Line (8) Electronic property: strong acceptor ‘A’, weak acceptor ‘a’, neutral ‘.’, weak donor ‘d’ and strong donor ‘D’. Line (9): topology again.  A window of width 16 residues is moved across the sequence from left to right, one residue at a time. At each position the different prop-erty-feature combinations (such as “charge-negative”, size “medium”) in the window are counted. The collection of these counts in a vector forms the feature at that position.  In the ex-ample shown above, the window width is shown as 16 residues. In the analyses, the width used for HMM modeling is 6 residues and that for NN modeling is 16 residues.  If the length of the protein is *L* residues, and window length is *l* residues, the number of feature vectors obtained is:* L*-*l*+1. The three shaded windows at positions 1, 23 and 50 have labels “completely non-TM”, “completely TM” and “mixed” correspondingly.   (**B) Feature vectors:** Feature vectors of the sequence corresponding to each of the window posi-tions are shown. The 10 rows of property number correspond to the Cij list of Eqn. 2. The win-dow position refers to the residue number of the first residue in the window.  Feature vectors cor-responding to the blue, red and yellow windows in (A) are shown in their corresponding color in the table. The class label of the feature vector is shown in the last row: completely nonmembrane -1, membrane 1.

The primary sequence of each protein is decomposed into five different sequences by replacing each amino acid with its property (see Figure 4A):

• Charge: 3 possible values: positive (H, K, R), negative (D, E), neutral (A, C, F, G, I, L, M, N, P, Q, S, T, V, W, Y)

• Polarity: 2 possible values: polar (C, D, E, H, K, N, Q, R, S, T, Y), nonpolar (A, F, G, I, L, M, P, V, W)

• Aromaticity: 3 possible values: aliphatic (I, L, V), aromatic (F, H,W, Y), neutral (A, C, D, E, G, K, M, N, P, Q, R, S, T)

• Size: 3 possible values: small (A, G, P, S), medium (D, N, T), large (C, E, F, H, I, K, L, M, Q, R, V, W, Y)

• Electronic property: 5 possible values: strong donor (A, D, E, P), weak donor (I, L, V), neutral (C, G, H, S, W), weak acceptor (F, M, Q, T, Y), strong acceptor (K, N, R)

The protein sequence representation at this stage has 5 rows of length L, where L is the length of the protein (Figure 4A). In other words, the residue *r*_*i *_at position *i*, is represented by its properties

*r*_*i *_= (*C*_*i*_   *P*_*i*_   *A*_*i*_   *S*_*i*_   *E*_*i*_)

where *C*_*i*_, *P*_*i*_, *A*_*i*_, *S*_*i *_and *E*_*i *_are the charge, polarity, aromaticity, size and electronic-property of the residue *r*_*i*_.

#### Step 2: Neighborhood analysis through a window

The protein sequence is analyzed with a moving window of length *l*; the window is moved along the sequence one residue at a time, each position of the window yielding a feature vector. The feature vector at position *i*, represented by *R*_*i *_is derived from the window beginning at the *i*^th ^residue and extending *l *residues to its right. It is given as

*R*_*i *_= [*C*_*ij*_]_1×10_

where, *C*_*ij *_is the count of property-value *j *in window *i*. The specific property-values counted by *C*_*ij*_'s are as follows:

C_i1_: count of "charge-positive"

C_i2_: count of "polarity-polar"

C_i3_: count of "polarity-nonpolar"

C_i4_: count of "aromaticity-aromatic"

C_i5_: count of "aromaticity-aliphatic"

C_i6_: count of "electronic property-strong acceptor"

C_i7_: count of "electronic property-strong donor"

C_i8_: count of "electronic property-acceptor"

C_i9_: count of "electronic property-donor"

C_i10_: count of "size-medium"

The choice of the above 10 properties is arrived at by studying histograms of number of segments versus percentage of residues of a given property in segments of length *l*, and identifying the properties that showed distinct peaks in the histogram for TM and non-TM segments (data not shown).

While r_i _is the vector of properties of the amino acid at position i, R_i _is the number of times a residue with a specific property value occurs in a window of length *l *starting at position *i *and extending to its right. When feature vectors R_i _are computed for every position of the window, moving to the right one residue at a time, the entire protein will have a matrix representation *P *(Equation 4), whose columns are the feature vectors

$$P={\left[\begin{array}{cccc} R_{1}^{T} & R_{2}^{T} & ... & R_{L-l+1}^{T} \end{array}\right]}_{\text{10 }\times \text{ L-l+1}}$$

$R_{i}^{T}$ is the transpose of vector *R*_*i*_. The number of rows in matrix *P *is 10, same as the length of the residue feature vector (Equation 3). Number of columns is *L-l+*1, where L is the length of the protein sequence and *l *is the window length. The columns contain the feature vectors corresponding to their positions in the protein sequence. The matrix *P *is referred to as the *protein feature matrix*. In Figure 4B, the columns excluding the class labels correspond to R_i_'s. The entire matrix excluding the class labels corresponds to P.

A protein feature matrix is created for each of the proteins in the dataset, both for training and testing purposes. The features extracted this way are used in topology modeling through HMMs and for feature classification based prediction by NNs.

### Singular value decomposition

Amino acid properties for feature representation (C_i1 _to C_i10_) are mutually dependent. It is therefore desirable to transform these feature vectors into an orthogonal dimensional space prior to use of these features for prediction. Such a feature selection process helps in biological interpretation of results since the differences between the classes of feature vectors can be visualized. Furthermore, this process reduces the number of parameters required to create the HMMs and NN.

To achieve this, protein feature matrices of all the proteins are concatenated to form a large matrix *A*, and subjected to singular value decomposition (SVD)

*A *= *USV*^*T*^

where *U *and *V *are the right and left singular matrices and S is a diagonal matrix whose elements are the eigenvalues of the matrix *P*. The feature vectors for analysis are now the column vectors of the matrix product [39]

*SV*^*T *^or *AU*^*T*^

Setting the diagonal elements of the last rows of S to zero is known to reduce noise in the representation of the feature vectors and also reduces over-fitting due to the small training set by the subsequent classifier to which the features are input. The eigenvalues (values of the principal diagonal in *S*) show the energy content in corresponding dimensions. The top 4 dimensions of S of our training data have been found to carry 85% of the energy (variance) and hence only these top 4 dimensions have been used for feature representation. The matrices *U*, *S *and *V *are dependent on the matrix *A *from which they are computed. Therefore, for each new protein, singular value decomposition should ideally be recomputed, but this would also require recomputation of all the statistical models built on the features derived trough singular value decomposition. To avoid this, the feature vectors along the same principal components can be approximated by multiplication *R*_*i *_with *U*^*T *^similarly as given in Equation. 6 [39].

### Hidden Markov Model topology modeling

#### Model architecture

**Figure 5:**
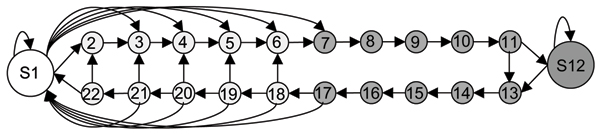
**HMM topology used for transmembrane prediction**. The architecture models cytoplasmic and extracellular regions in a single state S1 by 4 different feature clusters as a Gaussian mixture model. The interior membrane region, that is the TM segment excluding 5 residue positions each at both ends, is modeled by a single state S12 with 4 feature clusters as a Gaussian mixture model. The transition from non-TM to TM segment is modeled with 5 sequential states on the non-TM side and 5 sequential states on the TM side. States S18-S22 are connected to states S6-S2 respectively as shown, to accommodate short loops between two TM segments. States S11 is connected to S13 to allow accommodation of short TM helices. All the transition states, S2-S11 and S13-S22 are modeled with a single Gaussian feature cluster.

TMpro models the proteins with the simple and general architecture shown in Figure 5. The features extracted by TMpro, are derived from a window of residues, and are therefore *smoothed *among neighboring positions. Since the features in TMpro consist of simpler 2- or 3- rather than 20-valued properties, the feature distributions will vary less between adjacent residue positions. Hence, in the simplified HMM architecture self-transitions are allowed in the interior membrane region and in long loop regions. The architecture shown in Figure 5 is a model suitable for the topology of the known TM proteins, while being sufficiently flexible to accommodate possible new topologies. The window length *l *used for feature extraction is 6 residues.

#### Hidden Markov model parameter computation

In traditional *hidden *Markov models, the state transitions are unknown even for the training data. That is, the label, and hence the corresponding state in the HMM is not known at each observation instant. However, when modeling TM topology, the labels are known at the residue level. TMpro models the HMM states as follows: states S2–S11 and S13–S22 correspond to one residue position. State S7 marks the beginning of the TM segment and S17 its end. 5 residues on either end of the TM segments are numbered 7–11 and 13–17. The internal TM residues are numbered 12. The 5 residues of the non-TM region immediately preceding a TM segment are numbered backwards with respect to the TM segment as S6–S2, and the 5 residues following the TM segment are numbered forward S18–S22. Symmetrically positioned states on either side of S1 are tied to each other. For example, states S7–S11 and S13–S17, and states S2–S22 and S3–S21 are tied to each other. Where the loop length is short, the number of loop residues is shared equally between adjacent membrane segments, half being numbered in the S18–S22 series and half being numbered in the S6–S2 series.

Observation probabilities for each of the states are modeled by a Gaussian mixture model (GMM). The GMM of a state *S*_*i *_is computed by accumulating all those feature vectors whose residue labels are numbered *i*. For states S_1 _and S_12_, there are 4 mixtures in the model, and for all other states there is one Gaussian that models its feature space.

Transition probabilities and initial state-probabilities are computed by counting the number of corresponding labels of the sequences. Specifically, the probability of transition from state *i *to state *j *is given by

$$A_{i,j}=\frac{Count\;of\;sequence\;'ij'\;in\;the\;labels}{Count\;of\;'i'\;in\;the\;labels}$$

Similarly, the initial probability for state *i *is given by

$$\pi_{i}=\frac{Count\;of\;sequences\;where\;first\;label=i}{Number\;of\;training\;sequences}$$

In the training set there are no transitions from state 11 to state 13, because every TM segment is longer than 10 residues. However, to accommodate unseen topological models of TM proteins, for example that of aquaporin with two short helices of 8 residues length, a small mass of the transition probability from state 11 is assigned to state 13 and from state 10 to state 14.

#### Transmembrane helix prediction at residue level

To make a prediction of TM helix locations in a new protein, the protein is subjected to data preprocessing as during the training phase. The sequence of feature vectors is then presented to HMM and/or NN. Prediction of new protein topologies follows standard procedure [40]: the feature stream of the protein is evaluated by the hidden Markov model to arrive at a most-likely state sequence. Contiguous residues corresponding to the state labels 7–17 are considered a single TM helix segment in the final prediction. All other residues are labeled non-TM.

### Neural Networks for feature classification

Data preprocessing for NN is the same as for HMM. The window size (*l*) used is 16. During training, a class label is defined for each window based on the number of TM and non-TM residue labels in the window (Figure 4A):

• Completely-membrane (Class label = 1): If all residues in the window are labeled TM

• Completely-nonmembrane (Class label = -1): If all residues in the window are labeled non-TM

• Mixed (Class label = 0): If some residues in the window are labeled TM and some non-TM

#### Model architecture

**Figure 6:**
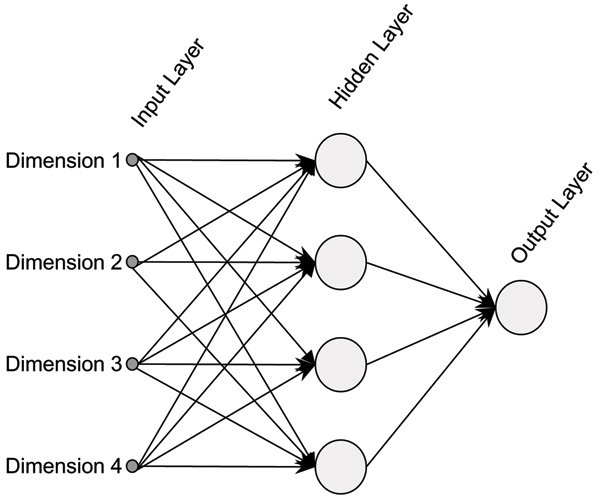
**Neural network architecture used for transmembrane feature classification**: The neural network has an input layer with 4 nodes that take each of the 4 dimensions of the feature vectors as input. The output layer has 1 tansig neuron that fires a value between -1 and +1 corresponding to non-transmembrane and transmembrane respectively. There is a hidden layer between input and output layers consisting of 4 neurons. The network is connected fully in the forward direction.

The number of input nodes of the NN is 4 and the number of output neurons is 1 (Figure 6). One hidden layer of 4 nodes is placed in between input and output layers (the choice of 4 units in the hidden layer is based on maximum accuracy in 10-fold cross validation of the training data). The model is fully connected in the forward direction. Each of the hidden and output neurons is a tansig classifier [41]. Each input dimension is normalized such that the range of all the dimensions is the same.

#### Model Training

The network is trained using back-propagation procedure [41], by presenting it with feature vectors and their corresponding target output class labels. Mixed label feature vectors are not presented for training, since they arise from both TM and non-TM residues and hence are expected to lie in the "confusable" region in the features space. The output neuron learns to fire -1 for non-TM features and +1 for TM features. For feature vectors that are ambiguous, the output lies in the range of -1 to +1. A threshold of 0.4 is chosen based on maximum accuracy in 10-fold cross validation of the training set to be used for automatic classification of the feature into its class.

#### Feature vector classification with NN

Each input feature vector causes the output neuron to fire an analog value ranging from -1 (non-TM class) to +1 (TM class). A threshold of 0.4 is used to label the residue at the first position in the window to be TM or non-TM. Since the feature is derived over a window of length 16, and threshold of 0.4 is "more confident" towards the TM label, the 8 residues starting from the first position of the window are all set to be of TM type (these numbers are derived through cross validation of the training set). The process is repeated for the next feature vector, and so on, and a TM label is assigned to 8 residues at a time every time the output neuron fires a value greater than the threshold.

### Implementation

#### SVD

Singular value decomposition of the protein feature matrix is computed using the SVDS tool in MATLAB^®^. Linear classification using Fischer's discriminant is carried out using the MATLAB^® ^statistical pattern recognition toolbox [42].

#### HMM and GMM implementation

Transition and initial probabilities of the model are computed as given by equations 7 and 8. Observation probabilities of a given state, which are Gaussian mixtures of the feature vectors assigned to that state, are computed with Netlab toolbox for MATLAB from the Nabney online resource [43]. The Bayes Net Toolbox (BNT) in a MATLAB^® ^environment obtained from the Murphy online resource is used for the task of predicting the state sequence of a test protein [44].

#### NN implementation

Training and classification procedures for neural networks are implemented using the Neural Net toolbox of MATLAB^®^.

## Competing interests

The authors declare that they have no competing interests.

## Authors' contributions

All four authors have contributed significantly to this work. MG has developed the algorithm, NB and RR provided direction for computational aspects of the algorithm and JKS provided insights into the biological/biophysical aspects of membrane protein structure. Manuscript has been prepared by MG and JKS and has been read and approved by NB and RR.

## References

[B1] Wallin E, von Heijne G (1998). Genome-wide analysis of integral membrane proteins from eubacterial, archaean, and eukaryotic organisms. Protein Sci.

[B2] Tseitin VM, Nikiforovich GV (1999). Isolated transmembrane helices arranged across a membrane: computational studies. Protein Eng.

[B3] Treutlein HR, Lemmon MA, Engleman DM, Brunger AT (1993). Simulation of helix association in membranes: modeling the glycophorin A transmembrane domain. System Sciences, 1993, Proceeding of the Twenty-Sixth Hawaii International Conference on: 1993.

[B4] Filizola M, Perez JJ, Carteni-Farina M (1998). BUNDLE: a program for building the transmembrane domains of G-protein-coupled receptors. J Comput Aided Mol Des.

[B5] Ott CM, Lingappa VR (2002). Integral membrane protein biosynthesis: why topology is hard to predict. J Cell Sci.

[B6] Kihara D, Shimizu T, Kanehisa M (1998). Prediction of membrane proteins based on classification of transmembrane segments. Protein Eng.

[B7] Sugiyama Y, Polulyakh N, Shimizu T (2003). Identification of transmembrane protein functions by binary topology patterns. Protein Eng.

[B8] Jayasinghe S, Hristova K, White SH (2001). Energetics, stability, and prediction of transmembrane helices. J Mol Biol.

[B9] White SH (1994). Global statistics of protein sequences: implications for the origin, evolution, and prediction of structure. Annu Rev Biophys Biomol Struct.

[B10] Kyte J, Doolittle RF (1982). A simple method for displaying the hydropathic character of a protein. J Mol Biol.

[B11] Sonnhammer EL, von Heijne G, Krogh A (1998). A hidden Markov model for predicting transmembrane helices in protein sequences. Proc Int Conf Intell Syst Mol Biol.

[B12] Tusnady GE, Simon I (1998). Principles governing amino acid composition of integral membrane proteins: application to topology prediction. J Mol Biol.

[B13] Fleishman SJ, Unger VM, Ben-Tal N (2006). Transmembrane protein structures without X-rays. Trends Biochem Sci.

[B14] Doyle DA, Cabral JaoM, Pfuetzner RA, Kuo A, Gulbis JM, Cohen SL, Chait BT, MacKinnon R (1998). The Structure of the Potassium Channel: Molecular Basis of K+ Conduction and Selectivity. Science.

[B15] Murata K, Mitsuoka K, Hirai T, Walz T, Agre P, Heymann JB, Engel A, Fujiyoshi Y (2000). Structural determinants of water permeation through aquaporin-1. Nature.

[B16] Chen CP, Kernytsky A, Rost B (2002). Transmembrane helix predictions revisited. Protein Sci.

[B17] Viklund H, Elofsson A (2004). Best alpha-helical transmembrane protein topology predictions are achieved using hidden Markov models and evolutionary information. Protein Sci.

[B18] Rost B, Casadio R, Fariselli P, Sander C (1995). Transmembrane helices predicted at 95% accuracy. Protein Sci.

[B19] Cao B, Porollo A, Adamczak R, Jarrell M, Meller J (2006). Enhanced recognition of protein transmembrane domains with prediction-based structural profiles. Bioinformatics.

[B20] Eyre TA, Partridge L, Thornton JM (2004). Computational analysis of alpha-helical membrane protein structure: implications for the prediction of 3D structural models. Protein Eng Des Sel.

[B21] Pilpel Y, Ben-Tal N, Lancet D (1999). kPROT: a knowledge-based scale for the propensity of residue orientation in transmembrane segments. Application to membrane protein structure prediction. J Mol Biol.

[B22] Ganapathiraju M, Klein-Seetharaman J, Balakrishnan N, Reddy R (2004). Characterization of protein secondary structure using latent semantic analysis. IEEE Signal Processing magazine.

[B23] Chen CP, Rost B (2002). Long membrane helices and short loops predicted less accurately. Protein Sci.

[B24] Ikeda M, Arai M, Lao DM, Shimizu T (2002). Transmembrane topology prediction methods: a re-assessment and improvement by a consensus method using a dataset of experimentally-characterized transmembrane topologies. In Silico Biol.

[B25] Melen K, Krogh A, von Heijne G (2003). Reliability measures for membrane protein topology prediction algorithms. J Mol Biol.

[B26] Cuthbertson JM, Doyle DA, Sansom MS (2005). Transmembrane helix prediction: a comparative evaluation and analysis. Protein Eng Des Sel.

[B27] Krogh A, Larsson B, von Heijne G, Sonnhammer EL (2001). Predicting transmembrane protein topology with a hidden Markov model: application to complete genomes. J Mol Biol.

[B28] Hurwitz N, Pellegrini-Calace M, Jones DT (2006). Towards genome-scale structure prediction for transmembrane proteins. Philos Trans R Soc Lond B Biol Sci.

[B29] Kernytsky A, Rost B (2003). Static benchmarking of membrane helix predictions. Nucleic Acids Res.

[B30] Hirokawa T, Boon-Chieng S, Mitaku S (1998). SOSUI: classification and secondary structure prediction system for membrane proteins. Bioinformatics.

[B31] Cserzo M, Wallin E, Simon I, von Heijne G, Elofsson A (1997). Prediction of transmembrane alpha-helices in prokaryotic membrane proteins: the dense alignment surface method. Protein Eng.

[B32] Cserzo M, Eisenhaber F, Eisenhaber B, Simon I (2004). TM or not TM: transmembrane protein prediction with low false positive rate using DAS-TMfilter. Bioinformatics.

[B33] Jayasinghe S, Hristova K, White SH (2001). MPtopo: A database of membrane protein topology. Protein Sci.

[B34] Tusnady GE, Dosztanyi Z, Simon I (2005). PDB_TM: selection and membrane localization of transmembrane proteins in the protein data bank. Nucleic Acids Res.

[B35] TMHMM 1.0 model. http://www.binf.ku.dk/~krogh/TMHMM/TMHMM1.0.model.

[B36] Landauer T, Foltx P, Laham D (1998). Introduction to Latent Semantic Analysis. Discourse Processes.

[B37] Rost B, Fariselli P, Casadio R (1996). Topology prediction for helical transmembrane proteins at 86% accuracy. Protein Sci.

[B38] Hollier MJ, Dimmock NJ (2005). The C-terminal tail of the gp41 transmembrane envelope glycoprotein of HIV-1 clades A, B, C, and D may exist in two conformations: an analysis of sequence, structure, and function. Virology.

[B39] Bellegarda J (2000). Exploiting Latent Semantic Information in Statistical Language Modeling. Proceedings of the IEEE.

[B40] Rabiner L, Juang B-H (1993). Fundamentals of Speech Recognition.

[B41] Haykin S (1998). Neural networks: A comprehensive foundation.

[B42] Franc V, Hlavac V (2004). Statistical pattern recognition toolbox for MATLAB. http://cmp.felk.cvut.cz/cmp/software/stprtool/index.html.

[B43] Nabney IT Netlab neural network toolbox:  (Electronic resourcce).

[B44] Murphy KB Bayes Net Toolbox for MATLAB,  (Electronic resource). http://www.cs.ubc.ca/~murphyk/Software/BNT/bnt.html.

